# Best evidence topic: Is ileocolic anastomotic leak rate higher in handsewn or stapler's anastomosis?

**DOI:** 10.1016/j.amsu.2020.11.075

**Published:** 2020-11-28

**Authors:** Sabry Abounozha, Adel Kheder, Talal Alshahri, Rashid Ibrahim

**Affiliations:** a Department of General Surgery, Northumbria Healthcare NHS Foundation Trust, Northumbria, UK; b Department of General Surgery, University Hospital Southampton NHS Foundation Trust, Southampton, UK; c Department of General Surgery, Imam Abdulrahman Alfaisal Hospital, Riyadh, UK; d Department of General Surgery, University Hospitals Plymouth NHS Trust, Plymouth, UK

**Keywords:** Anastomosis, Hands sewn, Ileocolic, Leaks, Staplers

## Abstract

A best evidence topic has been constructed using a described protocol. The three-part question addressed was: is ileocolic anastomotic leak rate higher in handsewn or stapler's anastomosis? Using the reported search, 150 papers were found. 6 studies were deemed to be suitable to answer the question. The outcomes assessed were anastomotic leaks rate in hands Sewn and stapler's ileocolic anastomosis. The evidence does not provide an agreed consensus for which modalities of anastomosis have higher anastomotic leaks rate. Until a high quality randomized control trial is performed, the authors recommend an individual approach in a term of selection of which anastomotic modalities to be used.

## 1 Introduction

This BET was devised using a framework outlined by the International Journal of Surgery [1]. This format was used because a preliminary literature search suggested that the available evidence is of insufficient quality to perform a meaningful meta-analysis. A BET provides evidence-based answers to common clinical questions, using a systematic approach of reviewing the literature.

## 2 Clinical scenario

You are a general surgery trainee. Assisting in a case of right hemicolectomy for cecal cancer, the consultant is about to perform the ileocolic anastomosis and he is quite concern about the possibility of leak, you are wondering which is the better modality to reduce the leak rate, hands Sewn or stapler's anastomosis?

## 3 Three-part question

In [patients planned for ileocolic anastomosis] is [the anastomotic leaks rate] is higher in [hands sewn or stapler's anastomosis]?

## 4 Search strategy

A. Embase 1974 to October 2020 using the OVID interface:

[ileocolic OR ileocolonic] AND [stapler OR staplers OR stapled] AND [hands Sewn OR hand Sewn] AND [anastomotic leak OR anastomotic leaks OR anastomosis leak OR anastomosis leaks].

B. Medline using the PubMed interface:

[ileocolic OR ileocolonic] AND [stapler OR staplers OR stapled] AND [hands Sewn OR hand Sewn] AND [anastomotic leak OR anastomotic leaks OR anastomosis leak OR anastomosis leaks].

The results were limited to English articles and human studies.

## 5 Search outcome

A total of 197 papers were found using OVID and 159 using the PubMed interface. A total of 150 papers were identified after we removed duplicates. Out of these 141 papers were excluded because they were irrelevant based on titles and abstracts. 9 full-text articles were screened and assessed for eligibility. From these, six papers were identified that provided the best evidence to answer the question eligible articles were defined as those articles that compared the anastomotic leak rate among patients who underwent ileocolic anastomosis with handsewn or stapler's techniques regardless of the indications for surgery. Up to our knowledge there is no universal consensus of a practical definition of ileocolic anastomosis yet available in the literature, so we relied on what was described by the authors as anastomotic leaks.

## 6 Result

**Table:** 

Author, date of publication, journal and country	Study type and level of evidence	Patient group	Outcomes	Key results	Additional comments
Zurbuchen et al. [2], 2012 Langenbecks Arch Surg Germany	Randomized controlled study, level II	The study included 67 patients who had ileo-colic resection for stenosing ileitis for Crohn's disease 36 side to side stapler anastomosis 31 end to end hand sewn anastomosis	Incidence of anastomotic leak between hand sewn and stapler anastomosis	No significant difference between the two groups in the incidence of anastomotic leakage. 0 of 36 in stapler group 2 of 31 (6.5%) in hand sewn group	Multicentre, randomized controlled trial, Small sample size Not mentioned clearly why the difference in incidence of anastomotic leaks is not significant All patients included in the study were known to have Crohn's disease Confounding factors are not mentioned (such as: comorbidity, BMI nutritional status, ASA score, and the complexity of the surgical procedure)
Nordholm-Carstensen et al. [3], 2018, Diseases of the Colon & Rectum, Denmark.	Retrospective Cohort Study, level III	The study included 1414 patients who had ileo-colic anastomosis after right hemicolectomy for Adenocarcinoma of the right colon. 391 (28%) had stapled anastomosis. 1023 (72%) had hand sewn anastomosis	Anastomotic leak rate between the 2 groups	21 leaks of 391 (5.4%) in the stapled group compared to 24 of 1023 (2.4%) in hand sewn group (*p* = 0.004)	Multicentre (nationwide), Large sample size, Multivariable logistic regression and propensity score matching were used to adjust for confounding Study included only cancer patients Retrospective No information on allocation to the stapled or handsewn anastomosis, A potential selection bias, did not mention how the leak was diagnosed
Jurowich et al. [4], 2018, BJS open, Germany	Retrospective Cohort Study, level III	The study included 4062 patients who undergone ileocolic anastomosis after right hemicolectomy for colon cancer. 2742 (67⋅5%) had a hand sewn 1320 (32⋅5%) had stapled anastomosis	Anastomotic leak rate between the 2 groups	No significant difference between the two groups. 106 anastomotic leaks in hand sewn group and 40 in stapler group. (3⋅9 *versus* 3⋅0%; *P* = 0⋅130)	Multicentre, large sample size, Univariable and multivariable analyses were performed Lack of information about the technique and materials used (e.g. end to end or side to side and type of sutures used) Study included only cancer patients, did not mention how the leak was diagnosed
Golda et al. [5], 2019, The American Journal of Surgery, Spain	Retrospective, Cohort Study, level III	The study included 470 patients who undergone primary ileocolic anastomosis after ileocecal resection, right and extended right colectomy for cancer. 234 patients (49.8%) had a hand sewn 236 patients (50.2%) had stapled anastomosis	Anastomotic leak rate between the 2 groups	No difference in anastomotic leak Between hands sewn and stapled anastomoses. 18 (7.6%) anastomotic leaks in hand sewn group 26 (11.0%) leaks in stapled group (P = 0.447)	Large sample size, Multivariate analysis, single centre Retrospective Study included only cancer patients, selection bias cannot be excluded
Gustafsson et al. [6], 2015, World J Surg, Sweden	Retrospective Cohort Study, level III	The study included 3428 patients who underwent Ileocolic Anastomosis after ileocecal resection or right-sided hemicolectomy for adenocarcinoma of the right colon. 1908 (55⋅7%) had a handsewn and 1520 (44⋅3%) had a stapled anastomosis	Comparing anastomotic leak between hand sewn and stapler anastomosis	The stapled anastomoses Had a higher leaks rate compared to the hand sewn. 36 leaks in stapled group Vs. 22 in hand sewn group (2.4 vs. 1.2%, p = 0.006)	Multicentre Large sample size, multivariate analysis, selection bias can't be excluded, Confounding factors are not mentioned (such as: comorbidity, BMI nutritional status, ASA score, and the complexity of the surgical procedure) Study included only cancer patients
Puleo et al. [7], 2012, Surg Today, Italy	retrospective cohort study, level III	The study included 999 patients who underwent ileocolic anastomosis after right hemicolectomy or ileocolic resection. 95.8% (957) of the patients were affected by cancer and 4.2% (42) were affected by inflammatory bowel disease, 46.4% (464) of the anastomoses were handsewn and 53.6% (535) were stapled	Anastomotic leak rate between the 2 groups	The rate of anastomotic leakage in cancer patients was higher in the handsewn group compared to the stapled group. 22 (4.9%) leaks in hand sewn group compared to 13 (2.5%) in stapled group (P = <0.05). The data for the Inflammatory bowel disease group were not statistically relevant	Multicentre, large sample size selection bias can't be excluded included both cancer and inflammatory bowel disease, Confounding factors are not mentioned (such as: comorbidity, BMI nutritional status, ASA score, and the complexity of the surgical procedure), did not mention the definition of the leak and how it was diagnosed

## 7 Discussion

Puleo et al. [7], conducted a large retrospective study in 2012 they included 999 patients who underwent ileocolic anastomosis for cancer and inflammatory bowel disease. 46.4% (464) of the anastomoses were handsewn and 53.6% (535) were stapled. The author concluded that the rate of anastomotic leakage among cancer patients was higher in the hand sewn group compared to the stapled group. 22 (4.9%) leaks in handsewn group compared to 13 (2.5%) in stapled group (P = <0.05). The incidence of anastomotic leak among patients with inflammatory bowel disease was not statistically significant.

In contrast, Gustafson et al. [6] in 2015, conducted a large multicentre retrospective study included 3428 cohorts who were diagnosed with right sided colon cancer and therefore underwent ileocolic anastomosis after a right hemicolectomy or ileocolic resection. The author concluded that the stapled anastomosis group had a statistically significant higher leakage rate compared to the handsewn group (2.4% vs. 1.2% P value = 0.006). Furthermore, Nordholm-Carstensen et al. [3], in 2018 reached the same conclusion after they conducted a large nationwide retrospective study which included 1414 patients who had ileo-colic anastomosis after right hemicolectomy for adenocarcinoma in the right colon. The author stated a 2-fold increase in anastomotic leak among stapled group versus handsewn group. There were 21 leaks (5.4%) in the stapled group compared to 24 (2.4%) in handsewn group (p = 0.004).

Nevertheless, despite these contradicting findings, another three large sized trials including randomised control trials showed no statically significant difference in anastomotic leak rate between handsewn and stapled techniques used for ileocolic anastomosis.

Those are the study which were conducted by Zurbuchen et al. [2] In 2012, which was a multicentre randomized controlled trial that included 67 patients who had ileocolic resection for Crohn's disease and also, the retrospective study in 2018 by Jurowich et al. [4] which included 4062 patients who underwent ileocolic anastomosis after right hemicolectomy for colon cancer and recently in 2019 Golda et al. [5]. Performed a single centre retrospective study including 470 patients who underwent ileocolic anastomosis after right colectomy for cancer.

### 7.1 Limitations of this review

1. Relatively weak level of evidence as there is only one randomized controlled trial out of the six studies included.
2. Lack of heterogeneity in the diagnosis, as some studies included cancer and some included Crohn's disease.
3. Lack of an agreed unified technique between the trials. As some used end to end and some side to side with different suturing materials and techniques which made it hard to compare.
4. Some of the papers have mentioned a possibility of selection bias between different centres and surgeons.

### 7.2 Clinical bottom line

There is insufficient scientific evidence to provide an answer regarding which modalities of anastomosis (handsewn or stapler's anastomosis) has higher anastomotic leaks rate in ileocolic anastomosis. Although from the evidence we have got, it seems that handsewn technique is more promising, as two studies compared to one study concluded that handsewn technique is associated with statistically significant lower leaks rate in comparison to stapler's technique. Until a large volume, multicentre, high quality randomized control trials can be performed, the author's advice a case by case individual approach in a term of selection of the anastomotic modalities, based on the skills of the surgeon and the availability of resources.

## Ethical approval

Not applicable.

## Sources of funding

None.

## Author contribution

SA: conducted the literature search and wrote the paper.

AK: assisted in the literature search and.

Writing of paper.

TA: assisted in writing of paper.

RI: assisted in the literature search, editing and Writing of paper.

## Provenance and peer review

Not commissioned, externally peer reviewed.

## Declaration of competing interest

None.
